# Detection of EGFR mutation in plasma using multiplex allele-specific PCR (MAS-PCR) and surface enhanced Raman spectroscopy

**DOI:** 10.1038/s41598-017-05050-4

**Published:** 2017-07-06

**Authors:** Xiaozhou Li, Tianyue Yang, Caesar Siqi Li, Deli Wang, Youtao Song, Lili Jin

**Affiliations:** 1 School of Science, Shenyang Ligong University, 110159 Shenyang, China; 2 College of Environmental Sciences, Liaoning University, 110036 Shenyang, China; 3 College of Medicine, Northeast Ohio Medical University, 44272 Rootstown, OH USA; 4 School of Life Science, Liaoning University, 110036 Shenyang, China

## Abstract

In this study, surface enhanced Raman spectroscopy (SERS) in combination with multiplexed polymerase chain reaction (PCR) was utilized to detect mutations of exons 19 and 21 of the epidermal growth factor receptor (EGFR) gene. Through the use of multiplexed PCR, the two mutation types were amplified in a single reaction. SERS was used on the PCR products to detect mutations. DNA mixtures with increasing mutation percentages showed good linear relationship between mutation rates and peak height. Then, this PCR-SERS method was used on the plasma of 48 patients with non-small cell lung cancer (NSCLC) to detect EGFR mutations. Analysis of variance (ANOVA) and receiver operating characteristic (ROC) analysis revealed that the peak height ratios were significant for identifying different mutation types. The specificity, sensitivity and accuracy obtained were all 100%. The proposed method was then validated through comparison with high resolution melting (HRM) and showed high concordance with HRM (Pearson correlation is 0.92). Finally, logistic regression was performed on EGFR mutation status and the clinical features of the 48 patients. Our study indicates that PCR-SERS is an effective, noninvasive, and economical method for the detection and monitoring of EGFR mutations in the plasma of patients with NSCLC.

## Introduction

Lung cancer is the most commonly diagnosed cancer and is responsible for 1.6 million deaths each year worldwide^1^. In China, alongside the deterioration in air quality, the mortality of lung cancer has increased by 465% over the past 30 years^2^. Among the total incidence of lung cancer, 80% of cases were non-small-cell lung cancer (NSCLC)^3^. Epigenetics, particularly research on DNA mutations, can provide important information that allows for a better understanding of lung cancer pathogenesis. Epidermal growth factor receptor (EGFR) mutations are key predictive factors for the efficacy of EGFR tyrosine kinase inhibitors in the treatment of patients with NSCLC^4^. The detection of EGFR mutations can assist targeted therapeutics and prognosis monitoring. On such a basis, methods that detect the presence of EGFR mutations at very low concentrations in a physiological environment are important. EGFR mutation testing methods include screening and targeted methods such as HPLC, HRMA, ARMS, pyrosequencing, and direct sequencing. However, none has yet been highly effective because of complicated procedures and long incubation periods^5^. Surface-enhanced Raman spectroscopy (SERS) has been recognized as an ideal analytical method for the analysis of biofluids. The merits of high enhancing factors of up to an order of 10–14 when compared to normal Raman spectroscopy, narrow Raman bands, and insensitivity to water lead to its wide applicability for the detection of various biological samples^6^.

In the last decade, the application of SERS on biomolecules has received much attention in clinical diagnosis. In particular, a variety of methods have been developed for analysis of DNA or gene mutations. There now exists direct detection of DNA sequence compositions^7, 8^, DNA structural modifications in cancer cells^9^, and of reactive oxygen species-induced DNA damage^10^.

Through adsorption on silver or gold nanoparticles or surfaces, Raman signals can be greatly enhanced. The SERS spectra of different nucleic acids or sequences show different feature peaks which can be used to differentiate the composition of that DNA. However, SERS is limited to short DNA sequences and is not suitable for the detection of cell-free DNA (cfDNA). The detection of point mutations of cfDNA in blood requires more sensitive approaches than ones used for the analysis of DNA extracted from tissues. For the SERS detection of DNA in blood or tissues, certain incubation and amplification methods are required. Those amplification methods include triple-helix molecular switch and enzyme-assisted amplification^11^, exponential strand displacement^12^, Taqman assay^13^, and ion-mediated cascade amplification^14^. Those methods all cleverly use the nanoparticle based nature of SERS and the amplification ability of biological engineering. However, those methods require a high concentration of genes which is difficult to obtain in the case of gene detection from plasma. Those methods additionally can only target single point mutations.

A method combining labelled primer multiplex PCR and SERS was introduced by Graham *et al*.^15^. The fluorescence labelled primers cause the PCR products to be labelled with fluorescence tags. Because of the difference in the length of PCR products and primers, only primers used for amplification were retained after purification by the final washing of unincorporated primers after the PCR. This method avoids the separation process, and can do multiplex genotyping by using the narrow bands of SERS.

Detection of gene mutations is mainly based on tissue biopsy, which is invasive, expensive, and time-consuming. cfDNA in Blood harbors similar biophysical properties as cancer cell DNA and can reflect the changes of cancer^16^. Detecting cfDNA in plasma requires high sensitivity methods and bimolecular amplification. Due to the narrow bands and sharp peaks of SERS techniques, SERS has been utilized to study multiplex detection of dye molecules and dye-labeled biomolecules such as DNA and proteins^17^. Multiplex detection of different DNA sequences can be achieved by distinct SERS peaks of dyes. As the fluorophores tend to have overlapping spectra, number of mutation types for the multiplex detection are limited.

Cell-free DNA molecules are circulating DNA sequences in plasma or serum and have been recognized as potential biomarkers for cancer. Various types of DNA modifications have been found in cfDNA, including mutations^16^. Tumour-related genetic and epigenetic alterations of cfDNA are relevant to cancer development^18^. Using cfDNA as detection target is noninvasive, readily available, and can be repeated multiple times which is important for prognosis monitoring of patients with NSCLC. Detecting circulating cfDNA in blood requires high sensitivity methods such as PCR, high-performance liquid chromatography (HPLC), sequencing, and others. Within this context, the PCR-SERS methodology offers a great promise for simplified, simultaneous, and sensitive detection of gene mutations in blood plasma.

In this paper, we explored the use of PCR-SERS for the detection of EGFR mutations in the plasma of patients with NSCLC. First, the PCR-SERS method was tested on two individual plasma samples, each containing either an EGFR mutation at exon 19 or at exon 21. Then, seven EGFR mutation mixtures with increasing mutation percentages were detected by PCR-SERS to see its detection limit. Finally, PCR-SERS was applied on samples from 48 patients with NSCLC. The results were verified by comparison to the high resolution melting (HRM) analysis. The two methods showed a good concordance (R = 0.92). Follow-up logistic regression analysis showed the correlation between clinical features of the 48 patients and EGFR mutation status. This study demonstrated that the PCR-SERS method provides a useful tool to detect gene mutations in blood plasma.

## Methods and Materials

### Materials

All chemicals used in this experiment were purchased at the highest grade from Sigma Aldrich. Fluorescence tagged primers were purchased from Sangon Biotech (China).

### DNA mixtures

Deletion mutations of exon 19 and exon 21 were diluted in wild-type (WT) DNA. Seven solutions were created which contained increasing percentages of mutated DNA- 0%, 1%, 10%, 20%, 50%, 80%, and 100% (0, 10^−12^, 10^−11^, 20^−11^, 50^−11^, 80^−11^, and 10^−10^ M) to test the detection limit and the ability for semi-quantitatively detecting mutation percentages of PCR-SERS method. The primers used for polymerase chain reaction (PCR) are 5′-GCATCGCTGGTAACATCCAC-3′ and 5′-AGATGAGCAGGGTCTAGAGC-3′ for exon 19, 5′-TGACCCTGAATTCGGATGCA-3′ and 5′-ATACAGCTAGTGGGAAGGCA-3′ for exon 21.

### Samples

This study was conducted in accordance with the Declaration of Helsinki. All patients provided written informed consent before sample collection. The Medical Research Ethics Committee of Shengjing Hospital approved all experimental protocols. Blood samples were obtained from 48 Chinese patients with NSCLC at Shengjing Hospital of China Medical University. All eligible patients had histologically confirmed NSCLC. Patient and cancer characteristics are detailed in Table 1. Patients were predominantly over the age of 40 (84%). About 50% of the patients (n = 24) had adeno carcinoma, versus 38% with squamous-type (n = 18), and 13% with large cell type (n = 6). Collected blood with added EDTA anticoagulent was centrifuged at 5,000 rotations/min for 10 min at 4 °C, and the plasma was collected in a 1.5 mL Eppendorf tube and stored at −80 °C. Plasma DNA was extracted with a QIAamp DNA Blood Mini Kit (Qiagen, Hilden, Germany) according to the manufacturer’s recommendations. The obtained DNA was stored at −20 °C.

**Table 1:** Clinical characteristics of the 48 NSCLC patients.

Clinical characteristic	Number
Gender	
Female	20 (42%)
Male	28 (58%)
Age	
≤39	8 (17%)
40–59	21 (44%)
≥60	19 (40%)
TNM stages	
I	5 (10%)
II	9 (19%)
III	16 (33%)
IV	18 (38%)
Tumor types	
Adeno	24 (50%)
Squamous	18 (38%)
Large cell	6 (13%)
Smoking status	
Non-smoker	22 (46%)
<20 package-year	22 (46%)
≥20 package-year	4 (8%)

### Multiplex PCR

Primers targeting EGFR exons 19 and 21 PCR were used in the multiplex PCR. Primer sequences and their attached fluorescence tags (R6G and Cy3) are listed in Table 2. Reactions were performed in a 1x PCR buffer containing 0.2 μM of each primer, 2.5 mM MgCl_2_, 0.3 mM dNTP, and 1.25 U DNA polymerase for a total volume of 25 μL. The PCR procedure involved a 5 minute initial denaturation at 95 °C, a 40-cycle amplification step (95 °C for 30 seconds, 59 °C for 60 seconds, and 72 °C for 60 seconds) and a final extension step of 72 °C for 5 minutes. After multiplex PCR amplification, the PCR products were purified to remove the unincorporated primers. For SERS analysis, PCR products were purified using the PCR purification kit (TIANGEN, China).

**Table 2:** Labeled primers used for the detection of mutations in EGFR.

EGFR gene	Primer sequence	Length (bp)
Exon 19	Forward: 5′-R6G-GCATCGCTGGTAACATCCAC-3′	290
	Reverse: 5′-AGATGAGCAGGGTCTAGAGC-3′	
Exon 21	Forward: 5′-Cy3-TGACCCTGAATTCGGATGCA-3′	302
	Reverse: 5′-ATACAGCTAGTGGGAAGGCA-3′	

### HRM

HRM was used on the same DNA mixtures and 48 plasma samples for comparison. HRM was performed on the ABI 7500 (Applied Biosystems, USA). Discussion about amplification of the target genes before the HRM process can be found in the PCR section. HRM was conducted immediately after the PCR: 95 °C for 45 s, 40 °C for 1 min, and a melt of 72 °C to 92 °C with one acquisition per 0.1 °C. EGFR mixtures with the four known mutation percentages (0%, 1%, 10%, and 100%) were used to test the detection limit of HRM. Normalized fluorescence of each DNA mixture was calculated and calibrated by an exponential curve using the R language. Normalized fluorescence of each sample was inserted in the calibration curve to estimate the mutation level.

### SERS

The SERS spectra were recorded by a Raman microspectrometer system (Renishaw, Britain) equipped with a He-Ne laser (λ = 632 nm) and an electrically cooled CCD detector. The laser power at the sample was 4 mW, and the accumulation time was 10 s. The spectra range was 400–1800 cm^−1^. Ag colloids were synthesized according to a modified Lee and Meisel procedure^19^. Silver nitrate (90 mg) was dissolved in distilled water (500 mL) at 45 °C and heated to near boiling under stirring. Then, sodium citrate solution was added (1%, 10 mL) and the solution was held at a gentle boil for 90 min with continuous stirring. The SERS measurements were done by mixing target DNA, Ag colloid and spermine (0.1 M) in a 1:1:1 volume ratio. The spermine acted as an aggregating agent and DNA backbone neutralizing agent^15^. The spectra were collected by focusing the laser directly onto the mixture.

### PCR-SERS

**Figure 1:**
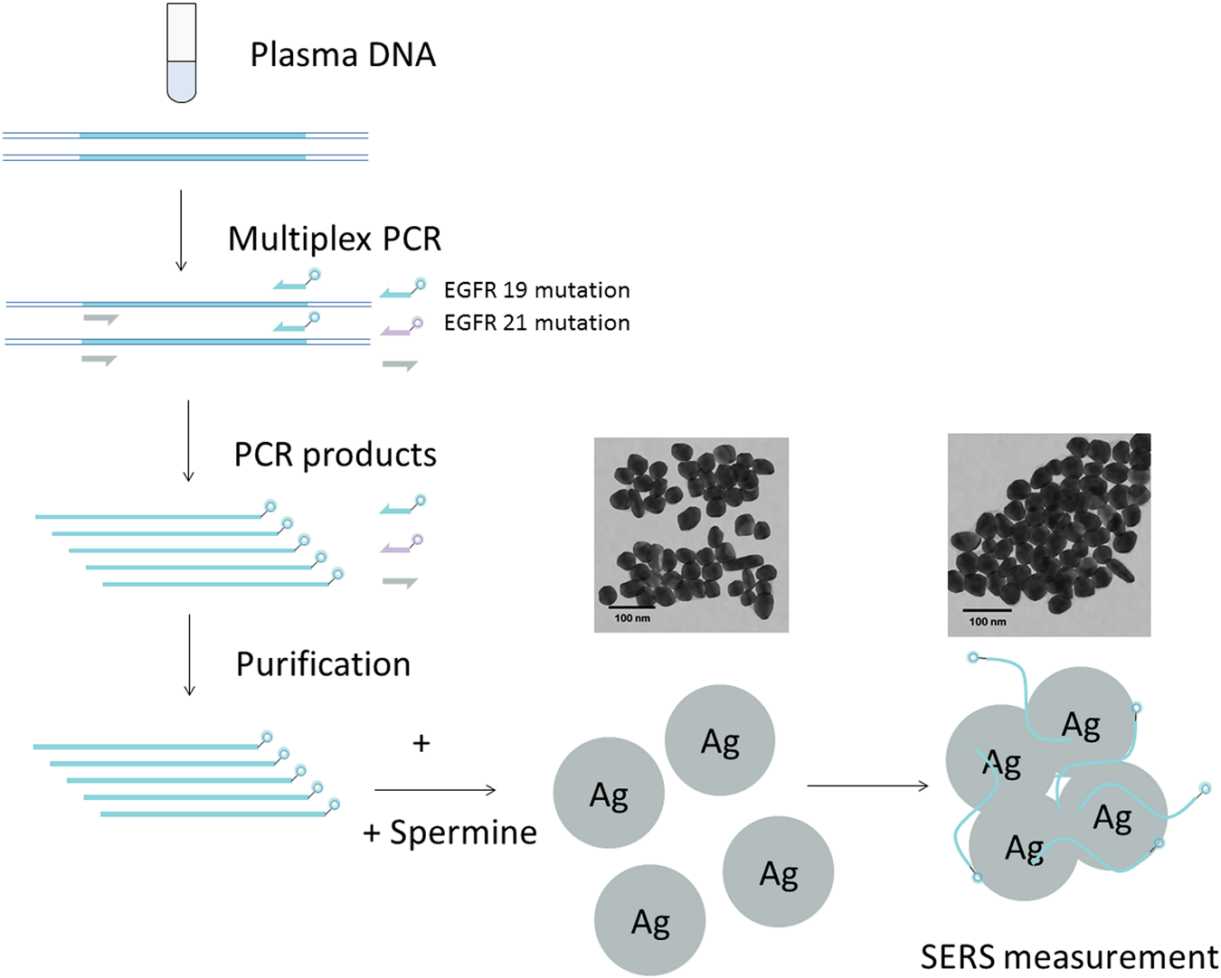
Schematic illustration of the PCR-SERS method for the detection of EGFR mutations in blood plasma.

As illustrated in Fig. 1, the PCR-SERS method involves three principal steps: (1) the PCR amplification of target mutated genes with labeled primers, (2) the purification process to remove unincorporated primers, and (3) the SERS detection of purified PCR products.

In the first step, two types of forward primers targeted two mutations in EGFR (either at exon 19, or exon 21), and each primer was labeled with a fluorescence tag at the 5′ end. This allowed the EGFR mutation genes to be amplified simultaneously while being labeled with the different fluorescence tags R6G and Cy3. The PCR products for the two pairs of primers were 290 bp and 302 bp in length. In the second step, the unincorporated labeled primers were removed with the purification kit. This process ensured that all retained dyes were labeled to mutated EGFR genes. In the third step, the resultant purified tagged PCR products were detected by SERS measurement. Due to the aggregating effect of spermine and PCR products, Ag nanoparticles aggregated together after the addition. As each dye was attached to one mutated EGFR gene, the SERS spectra of dyes reflected the existence of mutated EGFR genes.

### Statistical analysis

The interactions of gender, age, cancer stage, tumor type, and smoking status on mutation types of EGFR found in blood plasma were assessed by binomial logistic regression (BLR). The EGFR mutation statuses of the patients were used as output variables in the BLR analysis. One-way analysis of variance (ANOVA) was performed to determine the significance of the influence of each factor. The resulting odds ratios (OR) represent the odds that an outcome will occur under a certain condition, and thus reflects the association between clinical profiles of patients and EGFR mutation status in this paper^20^. OR values greater than 1 denote positive correlation and OR values less than 1 denote negative correlation. (According to the source cited, readers should be aware that an OR of >1 or <1 does not necessarily denote statistical significance, which is implied by the phrasing “positive/negative correlation”). Percentages of patients in the EGFR mutation positive and negative groups were calculated with 95% confidence intervals (CI). The clinical profiles included for analysis were gender, age, TNM stage, tumor type, and smoking status. All analyses were conducted using the R programming language (https://www.r-project.org).

## Results

### PCR-SERS method

**Figure 2:**
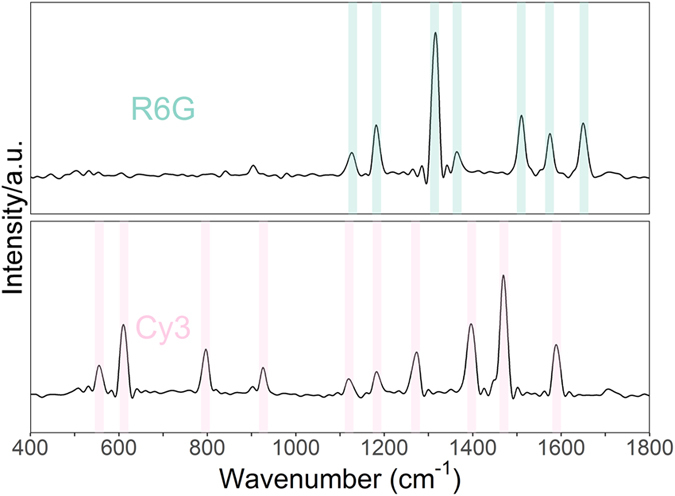
SERS of the 2 fluorescence tags.

For reference, SERS spectra of the two fluorescence tags were first measured. Two plasma samples with exon 19 and 21 mutations were taken from the 48 patients and tested by the proposed PCR-SERS method. Feature peaks of each tag are marked in colored rectangles in Fig. 2. The characteristic peaks of R6G were at 1129, 1183, 1314, 1365, 1510, 1574, and 1652 cm^−1^. The characteristic peaks of Cy3 were at 556, 612, 796, 927, 1121, 1184, 1271, 1398, 1471, and 1590 cm^−1^ (Fig. 2). The peak positions for each tag were different, but there was some overlap around 1120 and 1183 cm^−1^. The highest peak for R6G was at 1314 cm^−1^, while the highest peak for Cy3 was at 1471 cm^−1^. Thus, the SERS intensities at 1314 and 1471 cm^−1^ were used for discrimination analysis.

**Figure 3:**
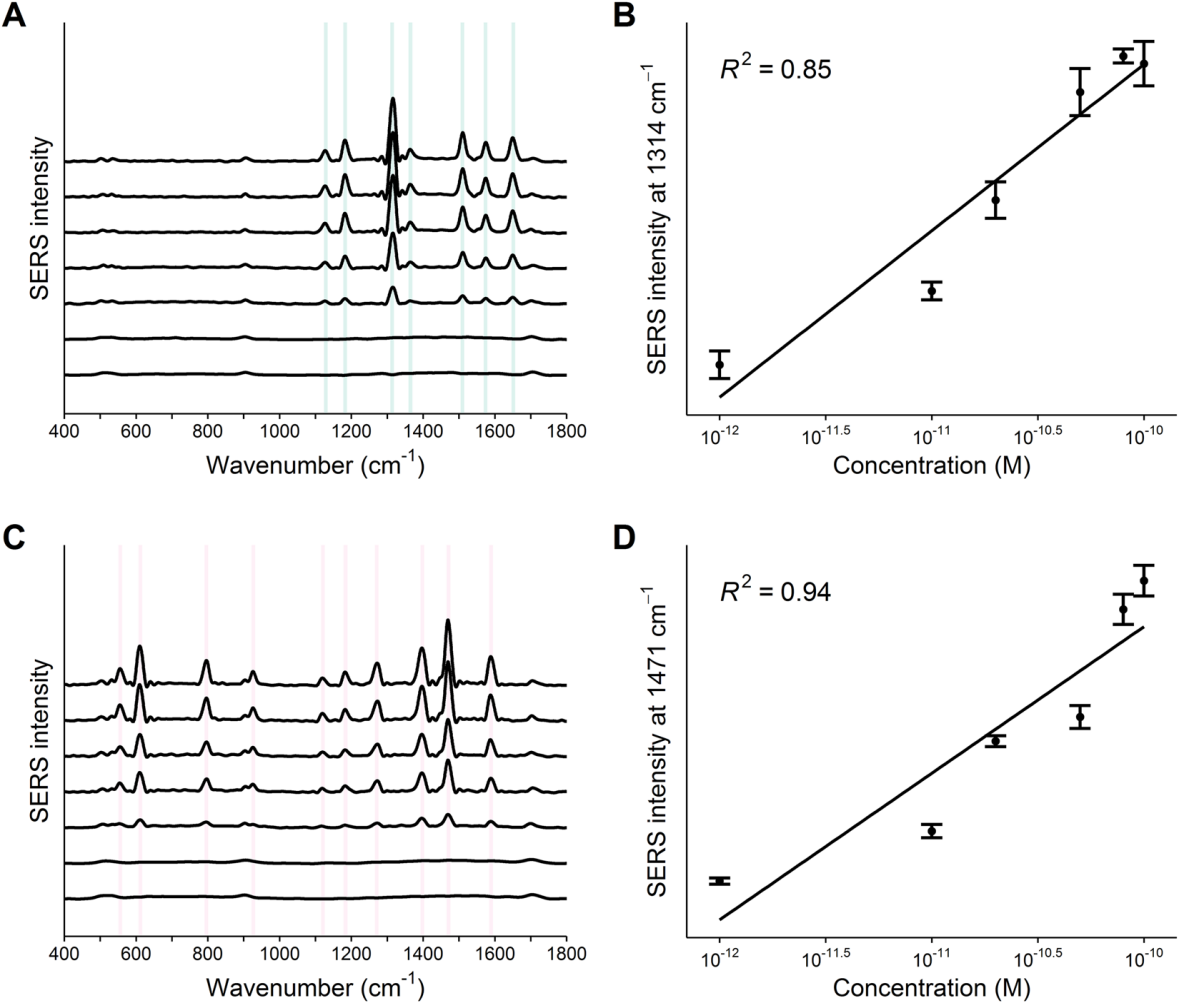
SERS spectra of PCR products of wild-type DNA mixed with increasing percentages of EGFR 19 (**A**) and 21 (**C**) mutations of 0%, 1%, 10%, 20%, 50%, 80%, and 100% (10^−12^, 10^−11^, 20^−11^, 50^−11^, 80^−11^, and 10^−10^ M). (**B**) and (**D**) are peak intensity changes at 1314 cm^−1^ and 1471 cm^−1^ for EGFR 19 and 21 separately with the increasing mutation percentages.

For quantitative analysis, we investigated the alterations in the SERS spectra of solutions with differing exon 19 and 21 mutation percentages separately. Seven DNA mixtures with the known mutation percentages of 0%, 1%, 10%, 20%, 50%, 80%, and 100% were prepared by mixing wild-type DNA with deletion mutated EGFR 19 and 21, respectively. As shown in Fig. 3, for both of the two mutations, the SERS signal improves as the EGFR mutation increases from 0% to 100%. For SERS spectra of 1%, characteristic peaks of R6G or Cy3 were unnoticeable. No significant SERS is observed in the spectra of 0%, indicating that the fluorescent R6G or Cy3 tag was not present, as expected, and that only EGFR mutation genes could cause SERS peaks. The Raman intensity at 1314 cm^−1^ and 1471 cm^−1^ was used for quantitative analysis for the mutation percentages of the two mutation types separately. As mutant EGFR concentrations increased, we observed that the Raman intensity of peak 1314 cm^−1^ and 1471 cm^−1^ increased accordingly. The detection limit were 5.97 × 10^−11^ M and 9.24 × 10^−12^ M for EGFR 19 and EGFR 21 respectively based on the standard deviation of the scatter plots.

**Figure 4:**
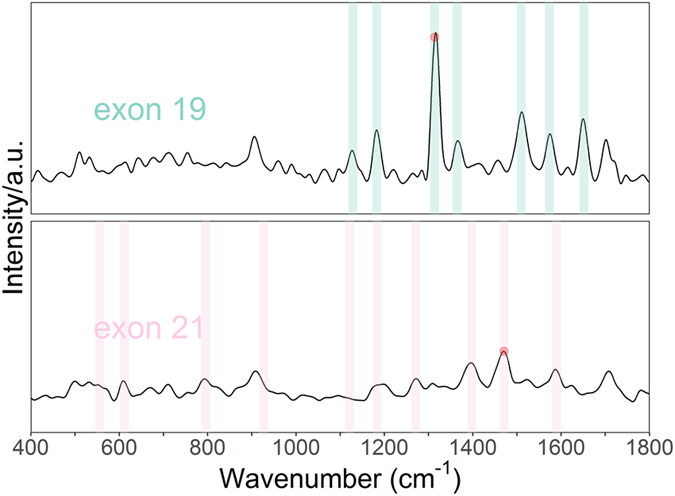
SERS of PCR products of human plasma having mutations at EGFR exon 19 and 21.

To demonstrate the feasibility of the proposed method for real sample analysis, two plasma samples of known EGFR mutations at exons 19 and 21 were investigated individually. As shown in Fig. 4, characteristic peaks of the SERS spectra of each dye (R6G and Cy3) can be clearly identified. Peaks of R6G are apparent, while peaks of Cy3 are much lower but still can be identified. The two apparent peaks at 1314 and 1471 cm^−1^ are still the highest in their own spectrum.

### Verification of samples

To briefly review the experimental procedure of this study, the plasma of 48 patients with NSCLC was examined through the use of multiplex PCR and SERS. Two types of products were obtained which corresponded to the two types of EGFR mutations under examination. The SERS spectra of these different PCR products showed the characteristic peaks of their associated fluorescent tags. To compare the peak heights of each tag, one highest peak of each tag spectra was chosen. Those peaks were 1314 cm^−1^ for R6G and 1471 cm^−1^ Cy3 (marked by a red point in Fig. 4). To judge the EGFR mutation position (exon 19 or 21), the peak heights of the two feature peaks at 1314 cm^−1^ and 1471 cm^−1^ were divided. The peak height ratio of H_1314_/H_1471_ is significant (p < 0.001). The specificity, sensitivity and accuracy obtained were all 100%. We can conclude that although the peaks are not visible because of the low percentage of corresponding tags, the height at the peak positions still had some changes contributed to them by those tags. Of the 48 unselected patients with NSCLC, we found that 11 patients (23%) had mutations in exon 19 and 10 patients (21%) had mutations in exon 21 of the EGFR gene.

**Figure 5:**
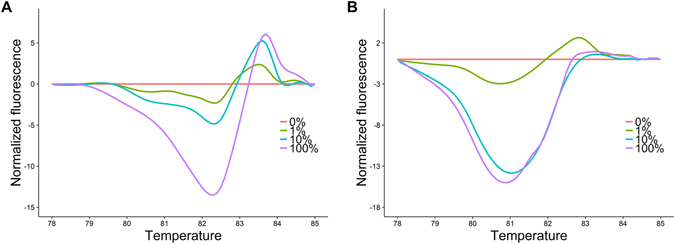
High-resolution melting analysis (HRM) with primers designed to detect mutations in epidermal growth factor receptor (EGFR) exon 19 (**A**) and exon 21 (**B**). DNA mixtures contained mutation rates of 0%, 1%, 10%, and 100%.

As verification, the same set of 48 samples was tested by HRM analysis. HRM used the same MIP primers as the above PCR-SERS to serve as better comparison between two methods. Figure 5 is the difference standard curve of HRM for the standardized mixtures of EGFR exon 19 mutations: 0%, 1%, 10%, and 100%. The HRM analysis results showed that 10 patients (21%) had EGFR an exon 19 mutation and 9 patients (19%) had an EGFR exon 21 mutation. Almost all mutations identified by PCR-SERS were correctly identified by HRM assays. However, PCR-SERS indicated more positive samples than HRM for both exons 19 and 21 (Table 3). Pearson correlation (R = 0.92) indicated a high degree of agreement between the two methods. Figure 5 illustrates the EGFR mutation state of exon 19 (Fig. 5A) and 21 (Fig. 5B) obtained from HRM.

**Table 3:** EGFR mutation predicted by PCR-SERS and HRM in 48 patients with NSCLC.

EGFR mutation	PCR-SERS	HRM
Wild-type	27 (56%)	29 (60%)
Exon 19	11 (23%)	10 (21%)
Exon 21	10 (21%)	9 (19%)

### Relationship between EGFR mutations and clinical profiles

The number of patients with plasma EGFR mutations in each clinical subgroup was counted and is summarized in Table 4. Among the 48 NSCLC patients, 44% (21 patients) had EGFR mutations of either exons 19 or 21 in plasma. Percentages of patients in TNM stages I, II, III, and IV who had either EGFR mutation were 40% (2 samples), 56% (5 samples), 38% (6 samples), and 44% (8 samples), respectively. The occurrence of EGFR mutations in adeno, squamous cell, and large cell lung cancer patients among our sample was 54% (13 samples), 28% (5 samples), and 50% (3 samples). A total of 8 (36%), 12 (55%), and 1 (25%) samples had EGFR mutation among our non-smoking, smoking less than 20 package-years (py) and smoking more than 20 py patient groups. To test the relationship of EGFR mutation status and clinical profiles of the NSCLC patients - gender, age, TNM stage, tumor type, and smoking status, binomial logistic regression (BLR) was applied on the sample set.

**Table 4:** Frequency of EGFR mutation in relation to clinical characteristics of NSCLC patients.

Clinical characteristic	Number	EGFR mutation (%)
Gender		
Female	20	11 (55%)
Male	28	10 (36%)
Age		
≤39	8	3 (38%)
40–59	21	9 (43%)
≥60	19	9 (47%)
TNM stages		
I	5	2 (40%)
II	9	5 (56%)
III	16	6 (38%)
IV	18	8 (44%)
Tumor types		
Adeno	24	13 (54%)
Squamous	18	5 (28%)
Large cell	6	3 (50%)
Smoking status		
Non-smoker	22	8 (36%)
<20 package-year	22	12 (55%)
≥20 package-year	4	1 (25%)

The EGFR mutation status was the output variable, and was divided into groups of “Yes” and “No”. The results of BLR reveal the odds ratio, 95% CI interval, and the p values of each comparison pair (Table 5). No significant association between the clinical characteristics (p > 0.05) and the status of EGFR mutations were found except for gender (p = 0.048, <0.05) and tumor type (p = 0.031, <0.05). Clinical profiles not significantly correlating with EGFR mutation frequency were age (p = 0.511 and 0.561), TNM stages (p = 0.625, 0.534 and 0.414), and smoking status (p = 0.262 and 0.387). These data suggested that NSCLC patients who are female or have adenocarcinoma have a higher incidence of EGFR mutation in plasma.

**Table 5:** Results of binomial logistic regression (BLR) on 48 NSCLC patients, describing the influence of gender, age, cancer stages, tumor types, and smoking status on the state of EGFR mutation.

	Odds ratio	95% Confidence interval		*P* value
		Lower bound	Upper bound	
Female vs male	0.41	0.16	0.98	0.048
Age < 39 vs 40–59	1.54	0.42	6.09	0.511
Age < 39 vs > 60	1.42	0.43	4.97	0.561
Stage I vs II	0.65	0.11	3.83	0.625
Stage I vs III	0.63	0.14	2.79	0.534
Stage I vs IV	0.53	0.11	2.43	0.414
Adeno vs large cell	0.66	0.18	2.37	0.524
Adeno vs squamous	0.34	0.12	0.88	0.031
Non-smoker vs py ≤ 19	1.67	0.69	4.09	0.262
Non-smoker vs py > 20	0.45	0.06	2.57	0.387

## Discussion

Identification of gene mutations is essential for personalized therapy in cancer. The advent of personalized therapy provides treatments with better efficacy and lower toxicity. In lung cancer, epigenetic alterations such as DNA mutations that lead to gene silencing are common events. EGFR mutations are considered to be an important predictive marker for the efficacy of EGFR tyrosine kinase inhibitor treatment. Currently, direct sequencing is used as the standard for detecting EGFR mutations, but it has limited sensitivity, high cost and long incubation times. Therefore, alternative methods for routine clinical gene mutation testing are required. A PCR-SERS approach for detecting EGFR mutation in plasma of patients with NSCLC is evaluated here. Utilizing the amplification of PCR and the high sensitivity of SERS, the PCR-SERS method can detect gene mutations in very low concentrations. We designed this study to determine whether PCR-SERS could be a diagnostically useful screening method for EGFR mutations in clinical blood plasma samples. First, PCR-SERS was tested on seven solutions with increasing EGFR mutation percentages on exon 19 and 21 separately (0%, 1%, 10%, 20%, 50%, 80%, and 100%). Figure 3 (A) and (C) is the resulting SERS spectra. The high sensitivity reached by our PCR-SERS method is based on the double amplification stage of PCR and SERS. The signal to noise ratio (S/N) was reduced on decreasing EGFR percentages. S/N was less than 4 below 1% mutation concentration and therefore we can assume such a percentage as the detection limit for the EGFR mutations by our method. The detection limit of PCR-SERS is very close to the one that can be reached using exponential strand displacement amplification (SDA)-based SERS approaches (1.4 pM)^12^.

Then, the PCR-SERS method was individually applied on plasma with known EGFR mutations at exon 19 and 21. In the PCR process, primers were labeled with fluorescence dyes to make the PCR products dye-labeled. The fact that the SERS spectra of EGFR mutations at different exons showed no characteristic peaks of dyes labeled to other exon proved the efficiency of the removal approach can be compared with the biotin-streptavidin capture process^15^. The SERS peaks of purified PCR products showed characteristic bands of tags R6G and Cy3, as would be expected from pure R6G and Cy3 as illustrated in Fig. 2. The peaks of R6G and Cy3 are apparent for the purified PCR products targeting EGFR mutations at exon 19 and 21. This experiment clearly showed the ability of PCR-SERS to detect low concentration gene mutations in biofluids such as plasma. Recent works report on DNA detection using SERS-based methods, employing specific binding or DNA sequences to “clamp” target mutated EGFR gene mutations on a chip^21^. These methods showed the versatility of SERS in combination with other biotechnology techniques. Nevertheless, the PCR-SERS methods used in this paper have the advantage of detecting concentrations as low as those found in blood plasma. Similar results were observed by using other PCR-like methods in combination with SERS^13, 14^. Our experiment has clearly showed that with the aid of PCR, multiplex SERS detection of EGFR mutations is sensitive enough to occur at concentrations low enough to include blood plasma.

In our experiment with PCR-SERS on the plasma samples of 48 NSCLC patients, we identified that 21 of the 48 samples had a mutation on either exon 19 or 21. Overall, 11 mutations were found in exon 19 and 10 mutations were found in exon 21. This overall mutation rate (21%) was much higher than reported EGFR mutation rates of European patients, but was similar to reported rates for East Asian populations. These results support reports that EGFR mutation rates of patients from East Asia are significantly higher than rates in Europe and North America^22^. Genetics, diet, and lifestyles could play a role. As shown in our comparison between PCR-SERS and HRM, the two methods were relatively comparable in their ability to detect EGFR mutations in human plasma.

In accordance with previously published results, the only statistically significant associations between our subgroups and the presence of EGFR mutations were gender and tumor type. EGFR mutations were detected more frequently in females than males and in adenocarcinomas compared to squamous cell carcinomas^23^. These results were consistent with our experiment.

Gene mutations play an important role in the pathogenesis of a wide range of cancers such as lung, colon and breast. Detections of genetic mutations can be used for the selection of treatments and in evaluating the response to therapies. The high detection accuracy of the PCR-SERS technique suggests that it could be used to detect and monitor gene mutations in the plasma of cancer patients. While we have only demonstrated 2-plex detection, the method could easily be developed to the simultaneous detection of more mutations. Future work can be done to expand the selection of dye labels to non-fluorescence dyes^24^. The key problem of this method currently is the availability of primers for multiplex PCR. One shortcoming of our method is that it can only detect known mutations. Nevertheless, it seems clear from the results presented here that PCR-SERS is a sensitive and simple method to detect gene mutations in blood plasma.

In conclusion, we have provided a “proof of concept” for the use of PCR-SERS as a high-sensitivity detection method of specific EGFR gene mutations in the plasma of patients with NSCLC. The method was successfully applied to the DNA mixtures with two EGFR mutations and plasma derived DNA of 48 patients with NSCLC. The specificity, sensitivity and accuracy of this method for discriminating two EGFR mutations and wild-type are all 100%. The suggested PCR-SERS method possesses several merits. First, it allows for the simultaneous detection of multiple gene mutations. Second, the high sensitivity is ensured by the amplification of PCR and enhancement of SERS. Third, the method can be expended to a wider range of gene mutations. By taking advantage of the high amplification ability of PCR and the intrinsically high signal enhancement of SERS, this method can sensitively measure gene mutations in blood plasma. Further logistic regression analysis in our experiment showed that the EGFR mutations under study were associated with gender and tumor type. We believe this PCR-SERS method is a promising tool for the multiplex detection of gene mutations in plasma or other biological samples.
